# A new hazard scenario at Vesuvius: deadly thermal impact of detached ash cloud surges in 79CE at Herculaneum

**DOI:** 10.1038/s41598-023-32623-3

**Published:** 2023-04-06

**Authors:** Alessandra Pensa, Guido Giordano, Sveva Corrado, Pier Paolo Petrone

**Affiliations:** 1 Science Department, Geology, University of Roma Tre, Largo S. Leonardo Murialdo 1, 00146 Rome, Italy; 2 ISPRA-The Italian Institute for Environmental Protection and Research, Via Vitaliano Brancati 48, 00144 Rome, Italy; 3 Department of Advanced Biomedical Sciences, Laboratory of Human Osteobiology and Forensic Anthropology, University Federico II of Naples, Via Pansini 5, 80131 Naples, Italy

**Keywords:** Natural hazards, Geology, Volcanology

## Abstract

Diluted pyroclastic density currents are capable to cause huge devastation and mortality around volcanoes, and temperature is a crucial parameter in assessing their lethal power. Reflectance analysis on carbonized wood from ancient Herculaneum allowed a new reconstruction of the thermal events that affected buildings and humans during the 79CE Vesuvius eruption. Here we show that the first PDC entered the town was a short-lived, ash cloud surge, with temperatures of 555–495 °C, capable of causing instant death of people, while leaving only a few decimeters of ash on ground, which we interpret as detached from high concentration currents. The subsequent pyroclastic currents that progressively buried the town were mostly higher concentration PDCs at lower temperatures, between 465 and 390 and 350–315 °C. Charcoal proved to be the only proxy capable of recording multiple, ephemeral extreme thermal events, thus revealing for the first time the real thermal impact of the 79CE eruption. The lethal impact documented for diluted PDC produced during ancient and recent volcanic eruptions suggests that such hazard deserves greater consideration at Vesuvius and elsewhere, especially the underestimated hazard associated with hot detached ash cloud surges, which, though short lived, may expose buildings to severe heat damages and people to death.

## Introduction

Diluted pyroclastic density currents are among the most lethal volcanic phenomena. They are highly turbulent ground hugging pyroclastic currents (PDCs), which can either originate at vent as dilute surges (especially during phreatomagmatic eruptions), or they can be associated with high concentration, valley confined basal undercurrents, from which they can detach and move independently, even across rough topographies, making their paths highly unpredictable^1–3^. Diluted PDCs are responsible for some of the deadliest volcanic disasters, such as that occurred on May 8, 1902 at St Pierre, Martinique, when nearly 30,000 people were instantaneously killed^4–6^, or on September 15, 1991 at Mt Unzen, Japan, which caused 44 fatalities^7,8^, or on November 5, 2010 at Merapi, Indonesia, where more than 200 people died^9,10^.

The main factors that cause casualties and injuries from diluted PDCs arise from a combination of (1) burnings due to their high temperatures^11–17^, (2) dynamic pressure^4,6^; (3) acidic gases injuries^18^, (4) asphyxia from ash inhalation^14,19^.

Due to their low density and turbulence, diluted PDCs are prone to fast mixing with ambient air, fast dissipating their initial temperature so that rarely diluted PDCs are associated with high temperatures. By contrast, diluted PDC enveloping high concentration flows, known as ash cloud surges^2,3^, can maintain very high temperatures as long as they are coupled with the high concentration basal flow^23^, which are instead thermally conservative^20–22^, and which continuously transfer upward both mass and thermal energy^11,23^. This implies that if and where detachment of the ash cloud surge occurs due to topographic effects (e.g.,^2,3^), even at distal reaches (e.g.,^14,24^), their initial temperature can be as high as the parent basal high concentration current.

However, once detached, dilute and turbulent ash cloud surges are short lived events, which very often leave on ground only few centimeters of ash before lifting-off, with very little potential of preservation in the geologic record, unless immediately and conservatively buried by other deposits of the same eruption (e.g., fall and/or non-erosional PC deposits). The little preservation potential has led to a limited number of studies on such kinds of deposits and related phenomena^1–3,5–7,25–28^, and possibly to an underestimation of ash cloud surges hazard, especially of their thermal impacts.

**Figure 1:**
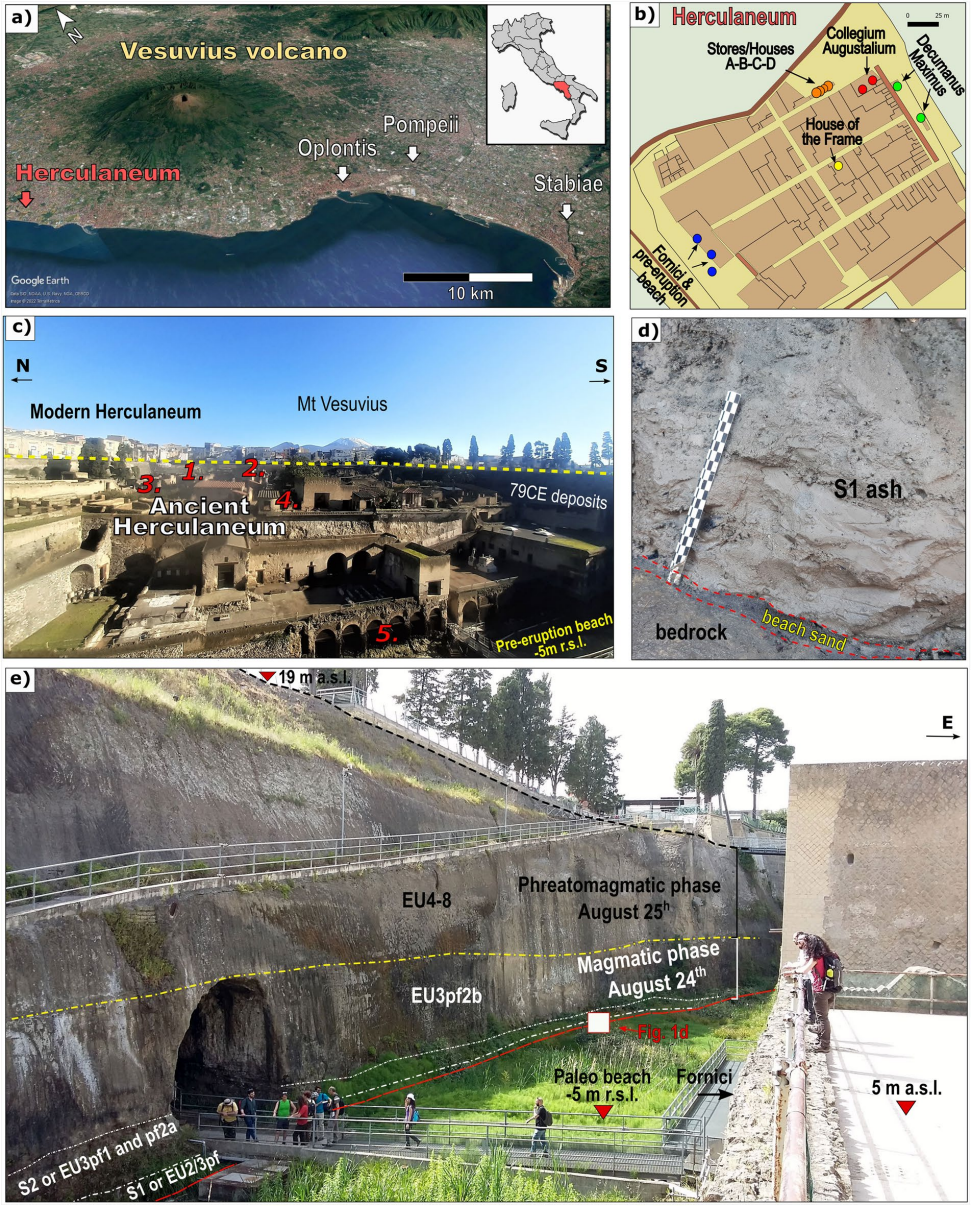
Herculaneum archaeological site location and the 79 CE Vesuvius eruption stratigraphy. (**a**) General map from Google Earth (Map data ©2022 TerraMetrics) of the Campanian plain with locations of Herculaneum, Oplontis, Pompeii and Stabiae archaeological sites; (**b**) ancient Herculaneum city map reporting the five sampling sites: Collegium Augustalium (red dots), Decumanus Maximus (green dots), Stores/Houses A-B-C-D (orange dots) located in the northern side of the city, House of the Frame (yellow dot) situated in the centre of the city, and Fornici & pre-eruption beach (blue dots) along the south-western side; (**c**) Panoramic eastward view of Herculaneum archaeological site; the dashed yellow line indicate the top of the 79CE pyroclastic deposits that buried the ancient town and onto which is built the modern city; the summit cone of Vesuvius (1281 m a.s.l.) is in the background; red numbers indicate the approximate location of sampling sites indicated in (**b**); (**d**) detail of the very base of the 79CE pyroclastic sequence; the S1 ash cloud surge deposits rest on few centimeters of black beach sand, in turn resting on the hard substrate; (**e**) Seaside (west) wall of the Herculaneum archaeological site showing the full stratigraphy of the 79CE Vesuvius eruption, annotated according to^30,31^; the triangle at 19 m a.s.l. is the viewpoint for (**c**).

The archaeological sites of Herculaneum and Pompeii are likely the most spectacular and famous historical cases of the interaction between pyroclastic currents, humans, and settlements (Fig. 1). Hundreds of human victims’ skeletons were found preserved within layers of pyroclastic deposits emplaced during the 79CE eruption of Mt Vesuvius^13–16,29^.

Despite the 79CE being one of the most studied eruptions, the exact timing, and the causes of death at Pompeii and Herculaneum are still debated, bearing implications for volcanological, archaeological and forensic anthropological studies.

At Herculaneum, the finding within a series of boatsheds and on the seashore of hundreds of skeletons embedded in the first ash surge deposit (known as S1^30^ or EU2/3pf^31^ Fig. 1e), coupled with the life-like stance of the victims’ corpses and the thermal-induced effects on skulls and bones, have been detected as evidence of intense heat exposure, sudden death and rapid disappearance of soft body tissues as a result of exposure of the skeleton to approximately 500 °C^13–16^. The unique finding of a vitrified brain from a skeleton still preserved in its original archaeological context on the site has also been reported as evidence of an early very high thermal event and subsequent rapid cooling of the early ash-cloud surge^17,32^.

Other authors proposed that the collagen preservation in some rib bones may indicate instead exposure to lower temperatures^33^. Therefore, the determination of the temperature and nature of the 79CE Vesuvius pyroclastic currents (PC) which killed the Herculaneum inhabitants are still a matter of debate, especially as the impact on human bodies, as well as on any other object, not only depends on the temperature of the PC, but also on its duration and rate of heat exchange. Geologic proxies for the emplacement temperatures of the PC sequence entombing Herculaneum have indicated so far average values around 350–400 °C; these are derived from the thermal remanent magnetization (TRM) of lithic clasts^34–36^ and reflectance of charcoal pieces extracted from PC deposits^37^. Both higher (up to > 450 °C) and lower (down to < 240 °C) temperature outliers have also been documented^13,17,36,37^, the latter especially where PCs interacted with edifices, either intact or collapsed, and seawater. However, none of the above-mentioned studies and methods have directly targeted the temperature of the poorly preserved early diluted PDCs that entered Herculaneum, instantly killed the people and barely buried their corpses^13–16^.

Here we explore the potential of reflectance analysis of charcoal formed during the progressive burial of Herculaneum in 79CE, to show the extreme high temperature impact of the very first ash cloud, which killed people and affected infrastructures. We will discuss how polymodal distributions of charcoal reflectance values, usually overlooked and smoothed by averaging values^10,22,37–44^, do instead record polyphase thermal events. In particular, given the non-retrograde nature of the carbonification process, short-lived, early high temperature events may be recorded by the incomplete charring of wood (i.e., disequilibrium carbonification which may affect only the outer part of timber^23^), and preserved if later burial occurred at lower temperature (i.e., equilibrium carbonification which can transform into charcoal the wood domains that were not already carbonized but that cannot overprint the reflectance attained by the charcoal domains formed at higher temperature^23^).

The Herculaneum archaeological site represents a unique case study to corroborate and test the validity and sensitiveness of charcoal reflectance as a geothermometer for both volcanological and bio-archaeological environments. The results may greatly contribute to bridge the gap among forensic, archaeological and volcanological studies carried out in the Herculaneum area, constraining the reconstruction of the principal lethal thermal event. This study highlights the thermal transport capacity of ephemeral diluted pyroclastic density currents, which are interpreted here as detached ash cloud surges, whose hazard is still largely underestimated.

The Roman towns of Herculaneum, Pompeii, Oplontis and Stabiae were devastated and buried during the 79CE Vesuvius Plinian eruption. The stratigraphy of this iconic eruption, described in two letters by the witness Pliny the Younger, has been reconstructed as corresponding to 8 main eruption units (^30,31,45–47^; Fig. 1e). According to Pliny the eruption occurred between August 24th and 25th. During the early phases, a sustained eruptive column rose to 30–33 km into the atmosphere^45^, emplacing phonolitic white pumice fall-out lapilli. Herculaneum was not affected by this early Plinian fall out phase, whose main dispersal axis was oriented SE towards Pompeii.

During the evening/night of August 24th, the pumice lapilli fallout changed to grey and phonotephritic in composition, and some pyroclastic currents formed due to partial collapses of the Plinian column. These early flows, recognized at Oplontis, Misenum, Stabiae and Herculaneum^46^, were described by Pliny the Younger as clouds "*travelling like streams over ground*"^48^. The associated PC deposits that form the basal units at Herculaneum have been named S1–S2^27^ or EU2/3pf and EU3pf^46^ (Fig. 1e). Here onward, we will refer to the basal unit as S1 and S2.

S1 deposits are made of 20–80 cm thick massive to stratified pale grey, coarse to fine ash^30^ (Fig. 1d). The dominant ash-size and reduced thickness of S1 were interpreted as evidence of Herculaneum being hit at first by a distal dilute pyroclastic surge^14^. The skeletons of about 350 people^16^, who sought shelter along the beach in twelve sea-front chambers, here after called Fornici (Fig. 1), and on the beach, were found lying within the S1 fine ash deposit. In town, almost completely abandoned by the Herculaneum citizens during the very early stages of the eruption, were discovered only a few dozen victims, as the case of that one found in the House of the Skeleton, those two in the Apodyterium^16^ and the one lying on a bed in the Collegium Augustalium^17^.

The unconsolidated S1 ash deposit is recognizable in only a very few places in the city. At the Fornici entrance (Fig. 1b, c) it ranges in thickness between 35 and 60 cm, whereas within the chambers it reaches 150 cm^30^.

Along the pre-eruption coastline, now at − 5 m below the present-day sea level (Fig. 1c, e), S1 lies directly on few cm thick black beach sand (Fig. 1d). Here S1 ranges from ca. 15 to 50 cm in thickness and contains numerous carbonized to partially carbonized wood pieces and rarer roof tiles^30^. S1 is overlain by ca. 70–100 cm thick massive layer made of ash and grey pumice lapilli embedding charcoal fragments and other archaeological material, which corresponds to layer S2^30^ (Fig. 1e). Human remains found along the beach were floating within the S1 fine ash deposit^14–16^. Above S1 and S2, the pyroclastic sequence is made by a 20 m-thick succession of massive, ash matrix supported lapilli tuff rich in lava and sedimentary lithics, deposited across the transition of the magmatic and phreatomagmatic phases of the eruption^30,31^ by high concentration PDCs which progressively buried the town (Fig. 1).

## Results

The reflectance is the optical property of charcoal to reflect the incident light and it increases at increasing degrees of carbonification^38,39,44,49,50^. We use charcoal from the Herculaneum archaeological site as proxy to reconstruct the thermal events that affected the town during the 79CE eruption. Carbonized wood fragments were collected from five different sites distributed along the flow path, from the most proximal northern side (Collegium Augustalium, Decumanus Maximus, and Stores/Houses A-B-C-D), central area (the House of the Frame) to distal southwest area along the pre-eruption beach (Fornici, Fig. 1b).

A total of 40 samples of charcoal fragments were sampled (see details in Supplementary Material Table S1): 12 from the Collegium Augustalium, 9 from the Stores/Houses A-B-C-D located along the upper Cardo III, 3 along the Decumanus Maximus, 1 in the House of the Frame along the upper Cardo II and 15 fragments at the Fornici/frontal beach. The charcoal fragments are both from shrubs and trees ripped off by the flow along the volcano flanks and manufactures (furniture and buildings). According to taxonomic studies^51^, the 90% of the timber employed for building houses and furniture at the time of the eruption by the Romans was conifer wood (fir wood^48^).

### Charcoal reflectance analysis

The measure of the reflectance (Ro) is a very robust and experimentally well constrained proxy for temperature at which the charcoal formed^38,39,50,52^. This method has been widely applied as geothermometer in different carbonification contexts: burial diagenetic environment, where organic material carbonizes slowly^44,53,54^, wild bush fires^49,55,56^, natural and anthropogenic fires^42,57^ volcanic deposits^10,22,23,36–38,40,41,58^. Despite such huge differences in carbonification processes, the treatment of reflectance data to retrieve paleotemperatures in volcanic environments has usually followed the same approach as for diagenetic charcoal, by the calculation of the mean reflectance value and related standard deviation, from fifty to one hundred of measures for each sample^10,22,23,36,37,41^. However, in volcanic environments, where short lived thermal events may not be conducive to complete carbonification, some authors have also proposed the use of the average of the three maximum reflectance values^40^ to better capture the disequilibrium carbonification at peak thermal conditions, with the obtained temperature still representing only a minimum as the heating duration process is unknown^38^. Recent studies have further shown that charcoal entombed in PC deposits may show polymodal distributions of reflectance data within individual samples and argued that different reflectance populations may instead record multiple thermal events experienced by the wood fragment^10,23^. This is possible as the process of carbonification is non-retrograde and requires timescales in the order of hours to be completed^38,50^. Therefore, a polymodal reflectance distribution can indicate the superposition of subsequent thermal events. To be preserved, these must be at progressive lower temperatures: (1) the early high temperature event(s) must be short lived so that wood can only be partially carbonized at high reflectance values, leaving other domains fresh; (2) the later lower temperature event(s) can then affect only the fresh domains conferring the polymodal distribution to charcoal reflectance in the single sample. It must be noted that if the later thermal event occurs instead at a temperature higher than the first one, the entire charcoal piece would be reset at higher T conditions, resulting in a unimodal population of reflectance values.

Based on these recent findings, in the data processing we will consider the presence of polymodal distributions to unravel he succession of subsequent thermal events, by identifying, in addition to the mean value and standard deviation, the main modes. The average of the three maximum Ro values (3Ro max) will be used to evaluate the minimum temperature of the maximum thermal event^40^.

**Figure 2:**
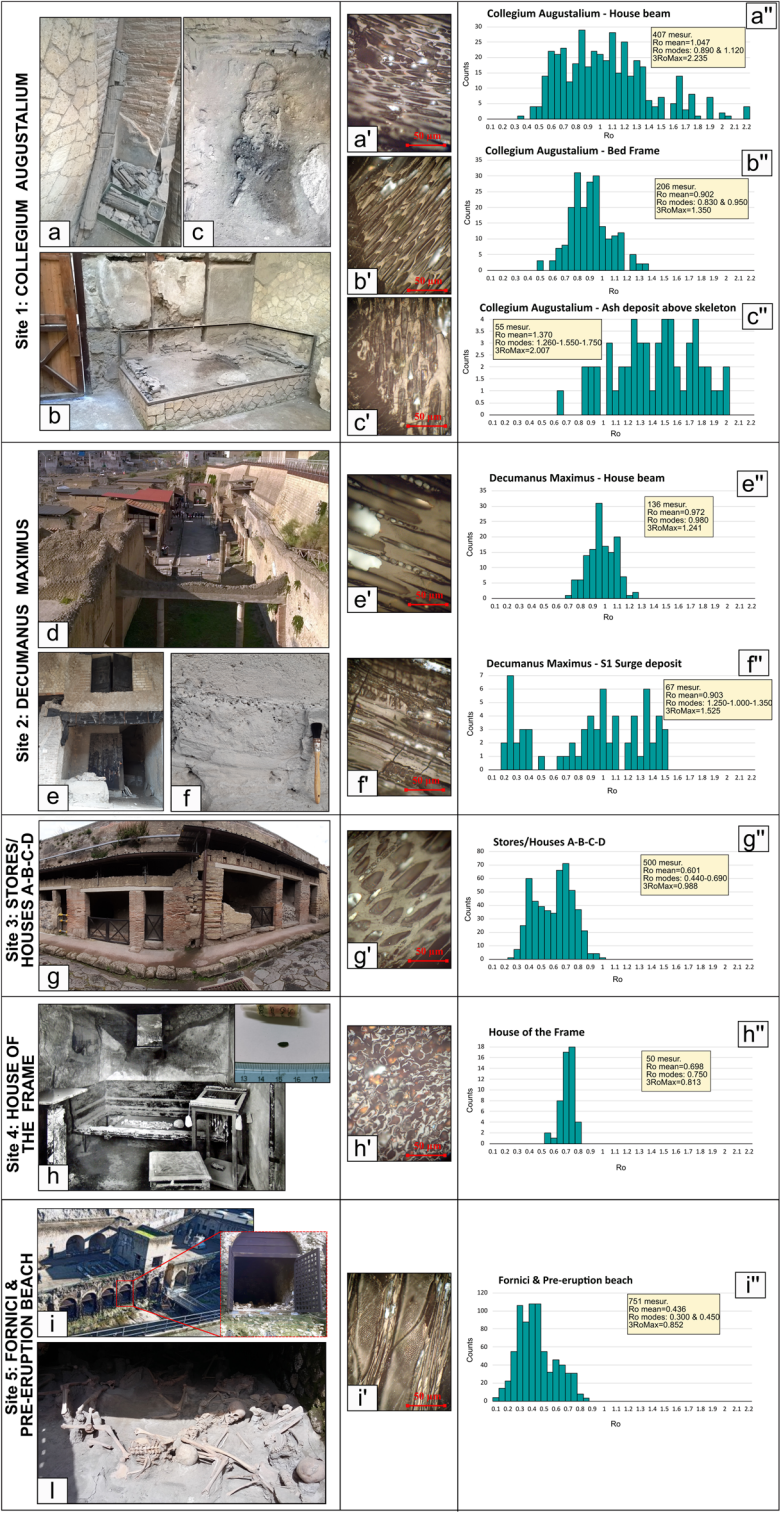
Carbonized wood fragments sampling site location and reflectance analysis results. Images from a to i show the locations where the carbonized wood fragments were collected (**a**—image of the support beam within the Collegium Augustalium **b**—photo of the carbonized bed frame within the Collegium Augustalium; **c**—photo of the remains of the man body lying on the bed when the eruption occurred; **d**—panoramic view of the Decumanus Maximus; **e**—carbonized house beam along the Decumanus Maximus and S1 ash deposit (in situ) ; **f**—particular of the S1 ash deposit outcrop along the Decumanus Maximus; **g**—view of the Stores/Houses A-B-C-D along the III Cardo Superiore; **h**—view of the House of the Frame and photo of carbonized seed recovered within the House of the Frame; **i**—panoramic view of the Fornici chambers located along the pre-eruption beach shoreline and **l—**photo of a group of skeletons found within one of the chambers. Microphotographs from **a′** to **i′** of charcoal samples under reflected light from polished stubs displaying absence of mineralization or alteration of the samples; Ro vs counts histograms indicated with letters **a′′**–**c′′, e′′**–**i′′**, displaying reflectance data distribution and Ro mean, modes and the mean of the 3 Ro maximum values.

All analyzed samples are not affected by post-depositional alteration nor remineralization (Fig. 2a′–i′). Reflectance data show both unimodal and polymodal distributions in different samples (Fig. 2a′′–i′′). Both trends have been described reporting in Fig. 2 and Table 1 the number of measurements performed on each sample, the mean reflectance, standard deviation, main modes, maximum reflectance value and the mean of the 3 maximum reflectance values. The values of the main modes and the dispersal of data around them are similar across the samples (Fig. 2 and Table 1). Data from samples belonging to the same wood source (e.g., bed frame or house beam) are presented together with a single frequency histogram. Results are described for the five sampling sites.

**Table 1:** Carbonized wood samples reflectance analysis data and temperature conversion.

	Sample source	Data Distrib.	N	Ro Mean	St. Dev	T °C Mean	Ro Mode	T °C Mode	Ro 3RoMax	T °C 3RoMax
Collegium Augustalium	7 frag. from support beam	Polym	407	1.047	0.365	442	0.890	421	2.235	555
							1.120	450		
	4 frag. from bed frame	Unim	206	0.902	0.159	422	0.830	412	1.354	477
							0.950	430		
	Frag. from ash above skeleton	Polym	55	1.370	0.326	480	1.260	465	2.007	537
							1.550	497		
							1.750	516		
Decumanus Maximus	2 frag. from house beam	Unim	136	0.972	0.112	432	0.980	433	1.244	465
	Frag. sieved from ash deposit	Polym	67	0.903	0.418	423	0.250	315	1.525	495
							1.000	436		
							1.350	477		
Stores/houses A-B-C-D	9 frag. from houses embedded within PC	Polym	500	0.601	0.153	377	0.440	350	0.988	434
							0.690	390		
House of the frame	Seeds	Unim	50	0.698	0.052	393	0.750	400	0.813	410
Fornici & pre-eruption beach	15 frag. from ash deposit within and outside Fornici and along the beach	Polym	751	0.436	0.154	350	0.300	325	0.852	415
							0.450	350		

Most of the sampled charcoal comes from sites that were fully excavated in the 1960s, and at that time the volcanic deposits stratigraphy was not documented. Therefore, some samples cannot be precisely located within the stratigraphy of the eruption. However, the samples were collected from the first 1–2 m above the ground and than, based on the stratigraphic sequences still preserved on the site, they can be referred to the S1–S2 deposits, and possibly the unit above (Fig. 1). In the following section we will detail otherwise only for those sites where samples can be associated with a specific volcanic unit.

#### Site 1: Collegium Augustalium

668 reflectance measurements were made on 12 charcoal samples taken from the Collegium Augustalium: 7 samples from a roof support beam in use during the restauration of structures following the 62 AD earthquake, work in progress at the time of the 79 AD eruption (Fig. 2a), 4 samples from a bed frame (Fig. 2b), and one sample from the ash deposit covering the body remains of a man found lying on the bed (Fig. 2c)^17^ (See Supplementary Material Table S1). The ash covering the body and the wooden bed is rich in grey pumice lapilli, the comparison of which with similar stratigraphic sequences found in the town suggests S2 as the best attribution. Samples from the support beam and ash deposit above the skeleton show polymodal data distribution (Fig. 2a′′, c′′) with two and three main modes, respectively. The support beam fragments analyzed display mean reflectance (Ro mean) of 1.047 (STDEV = 0.365) and two main modes at Ro = 0.890 and 1.120. The mean of the 3 maximum reflectance values (3Ro max) is 2.235. The charcoal from the ash covering the man’s body infers Ro mean data of 1.370 (STDEV. = 0.326) and three main modes at Ro = 1.260, 1.550 and 1.750. The mean of the 3Ro max is 2.007. Charcoal fragments from the bed frame show about unimodal data distribution (Fig. 2b′) with Ro mean corresponding to 0.902 (STDEV = 0.159). Due to its unimodal data distribution the two main modes 0.830 and 0.950 are very close to the Ro mean value. The mean of the 3Ro max is 1.354.

#### Site 2: Decumanus Maximus

Charcoal fragments collected along the Decumanus Maximus (Fig. 2d) belong to a house beam (two samples, Fig. 2e), located in front of the Collegium Augustalium, and to a preserved stratified fine ash deposit resting directly on the ground (one sample, Fig. 2f) attributable to S1. In consideration of its elevation above the ground, the house beam was presumably buried within the lapilli tuff succession that buried the town (EU3pf2b and EU4-8 in Fig. 1). Fragments from the center and from the rim of the house beam were analyzed to compare the carbonification degree; whereas multiple charcoal bits were extracted from the collected fine ash deposit (S1 or EU2/3pf in Fig. 1e).

The results of the performed 136 reflectance measurements indicated unimodal data distribution for the house beam sample, with reflectance values ranging from 0.500 to 1.350. The Ro mean value corresponds to 0.972 (STDEV = 1.112) and the main mode value of 0.980 is almost identical to the Ro mean value. The mean of the 3 Ro max is 1.244. The 67 reflectance measurements performed of carbonized wood bits retrieved from the ash deposit indicate polymodal distribution with three distinct modes. The Ro mean value is 0.903 (STDEV = 0.418), while the identified modes correspond to 0.250, 1.000 and 1.350. The mean of the 3Ro max is 1.525.

It must be noted that the samples belonging to the core and the rim of the house beam (ERC-54A and ERC-54B Supplementary Material Table S1) show the same carbonization degree.

#### Site 3: Stores/Houses A-B-C-D

A total of 500 reflectance measurements were carried out on the nine charcoal samples collected within the Stores/Houses A-B-C-D located along the NNW side of the city (Fig. 2g). These charcoal fragments belong to the floor beams on the second floor of these Stores/Houses (Supplementary Material Table S1).

Reflectance data results of the four Stores/Houses were merged into a single frequency histogram (Fig. 2g′′). Data distribution clearly shows a polymodal trend with two clear modes. The Ro mean is equivalent to 0.601 (STDEV = 0.153), the two identified modes correspond to 0.440 and 0.690, whereases the mean of the 3 Ro max is 0.988.

#### Site 4: House of the frame

The House of the Frame is located in the middle of the city along the upper Cardo II (Fig. 1b). Carbonized seeds, belonging to still unidentified plant, were found inside the house, associated to the skeletal remains of a child (Fig. 2h). As evidenced by macroscopic morphological analysis of the fine ash filling the child’s skull and its inclusions, as also testified by CT scans performed on it, this fine ash deposit is similar to the one in which the skeletal remains of the man were found within the Collegium Augustalium, so that this young victim, died instantly on arrival of S1 like all other Herculaneum residents^16^, was likely engulfed and buried by S2. The fifty reflectance measurements carried on one seed indicate a unimodal data distribution (Fig. 2h′), concentrated in a narrow reflectance range between 0.550 and 0.800. The Ro mean corresponds to 0.698 (STDEV = 0.052), the main mode is 0.750, very close to the Ro mean value; the mean of the 3Ro max is 0.813.

#### Site 5: Fornici/ Pre-eruption beach

The 15 charcoal samples collected within the Fornici and along the pre-eruption coastal beach (Fig. 2i) display both complete and partial carbonification (Supplementary Material Table S1). Charred wood samples were extracted from the S1 ash deposit where the victims’ corpses were unearthed (Fig. 1e). The 751 reflectance measurements indicate a polymodal data distribution with two main modes. The Ro mean value determined in this site is the lowest respect with the other sampling sites, corresponding to 0.436 (STDEV = 0.154). The two main modes identified are 0.300 and 0.450, while the mean of the 3Ro max is 0.852.

### Temperature conversion

Pyrolysis experiments using different plants and heating protocols have produced several curves that correlate the increasing temperature and charcoal reflectance^38,39,42–44,49,50,55,56^. These pyrolysis curves have similar trends, but differences do occur (See Supplementary Material Text S1, Fig. S1, Table S2). Based on the species of wood carbonized and on the investigated process of carbonification associated with pyroclastic currents, our temperature estimates refer to the curve by^55^ (See Supplementary Material Table S2 and the flow diagram in Fig. S2).

Table 1 shows the conversion of reflectance values into temperature. Due to the polymodal distribution displayed by almost all the analyzed samples we consider the Ro main modes, rather than the Ro means, as the most representative reflectance values of each sample. Furthermore, we use the 3Ro max values measured, for the evaluation of the minimum temperature of the highest thermal event^40^.

The highest temperatures were detected in the northern part of the city at Collegium Augustalium and along the Decumanus Maximus, where the 3Ro max values of charcoal show a temperature range between 495 and 555 °C.

The Ro modes between 0.690 and 1.260 recognisable both in the northern and in the southern-west side of the city, show decreasing temperature from 465 to 390 °C (Table 1).

The lowest Ro modes ranging from 0.250 and 0.450 were identified at Decumanus Maximus and Stores/Houses A-B-C-D but predominantly in charcoal samples collected at the Fornici and along the pre-eruption beach. The relative temperatures range from 315 to 350 °C. In Table 1 reflectance values converted into temperature and organized by sampling site are reported in detail.

In the table are reported in detail the results obtained from the reflectance analysis performed on the 40 samples collected at Herculaneum divided by sample site. Sample source, data distribution (unimodal or polymodal), number of fragments measured (N), Ro mean value, standard deviation, Ro modes and mean of the 3Ro maximum values measured and relative temperature conversion, using^55^ pyrolysis curve, are reported.

## Discussion

Diluted pyroclastic density currents have been fatal during numerous volcanic events in history. Despite the numerous episodes, low concentration PDCs hazard is still underestimated and not fully contemplated in hazard maps. According to^2^ the vulnerable zone should be double extended respect to the associated basal high concentration pyroclastic currents.

The under-consideration of the hazard of such turbulent, dilute, and high temperature pyroclastic currents, lies in the little preservation in the stratigraphic record of their thin and easy to wash-away deposits despite their high thermal impact on people and objects.

The recent volcanic episodes occurred in 1991 at Unzen^7,8,26,59^, in 2010 at Merapi Volcano^9,12^, in 2018 in Guatemala^60,61^, and in 2019 in New Zealand^62,63^ highlight the need to deepen our understanding of diluted PDCs hazard in terms of their thermal impact^11^.

In the case of Fuego de Guatemala 2018 eruption when more than 300 people died (although independent evaluations sadly suggest up to 2900 deaths)^11,60^, low concentration overriding ash cloud surges detached from valley confined high concentration flows killing many of them for suffocation and severe burns^60^. Some victims’ bodies were found on topographic highs, far from the valley ponds where thick and high concentration pyroclastic currents accumulated, and they were only partly covered by thin ash layers displaying pugilistic attitude as a result of exposure to high temperatures^60^. They were therefore certainly killed by the high temperature of the turbulent ash cloud detached at the periphery of high concentration flows, rather than by the impact of dynamic pressure. Similar was the case of the detached ash cloud surge occurred on September 15, 1991 at Mt Unzen, Japan, which caused 44 fatalities^7,8^ on a high relief bounding an incised valley where the high concentration PDC kept being confined. These tragic volcanic events display remarkable similarities with the most iconic eruption of the 79CE Vesuvius.

The heat-induced effects suffered by the victims, notably the explosion and charring of skulls, vaporization of brains, cracked and charred bones, cracked teeth, contraction of limbs and thermal degradation of blood haemoproteins^14–16^ indicate the occurrence of an early extremely high thermal event higher than the previously estimated temperature of about 500 °C. Unlike Pompeii, where many bodies show the typical post-mortem stance known as pugilistic attitude, the lack of such corpse attitude at Herculaneum testifies to the rapid disappearance of soft tissue, as the pugilistic stance is due to dehydration and shortening of muscles induced by intense heat^14,16^. However, until now, no direct measures of such high temperature early PC event were made at Herculaneum.

Our study on charcoal reflectance records for the first time the occurrence of subsequent thermal events at decreasing temperature which affected Herculaneum. By comparing the pyroclastic stratigraphy (Fig. 1e) and the thermal stratigraphy recorded by the polymodal distribution of charcoal reflectance we can reconstruct the succession of PC events that impacted the city.

The first diluted PDC (S1 or EU2/3pf according to authors) entered in Herculaneum with a temperature exceeding 550 °C recorded by samples collected at the Collegium Augustalium and the Decumanus Maximus (Table 1). This is the minimum temperature of the thermal event as the polymodal charcoal reflectance records an early uncomplete carbonification which testifies a short-lived event, unable to reach full equilibrium, which in experiments is usually attained after at least 24 h, at constant temperature, for pieces of wood measuring 2 × 5 cm^38^.

This early > 550 °C event was later followed by the succession of PCs which finally buried the town under 20 m thick volcanic deposits (Fig. 1e). These later flows were characterized by lower temperatures as testified by the presence of multiple modes within the same charcoal sample from which we inferred at least two carbonization events at temperatures ranging from 390 to 465 °C and from 315 to 350 °C, respectively. The lower temperatures of these later events can be explained by the progressive involvement of ground water during the course of the eruption (see phreatomagmatic phase^31^; Fig. 1e).

The occurrence of an early > 550 °C short lived diluted PDC event leaving only a thin ash layer on the ground, and later followed by the deposition of lower temperature but thicker pyroclastic deposits, allows to understand the conditions for the formation and preservation of a vitrified brain recently discovered within a victim’s skull in the Collegium Augustalium^17^. The transformation into glass of fresh cerebral tissue in a hot environment is only possible if two conditions are met: (1) the heating event is short-lived, so that the tissue is not fully vaporized^15^, and (2) once the diluted PDC has vanished, the body is not fully entombed in a hot deposit, a necessary condition to allow the very rapid cooling required to attain vitrification^17,66^. This allows to recognize that S1 was an ephemeral, extremely hot, dilute event, and that a sufficient time interval occurred for the fast cooling of the body still partly exposed to air before the following PCs progressively entered and covered the town. The lower temperature of these later PC deposits explains the preservation of the vitrified brain, as well as of the high reflectance values within polymodal distributions. If subsequent PC were at higher temperatures, the vitrified brain would have been reheated above the glass transition temperature and gone lost in its neuronal ultrastructure, which is instead integrally preserved^32^, as well as the charcoal fragments would had been totally reset at higher, unimodal Ro values.

The temperature of S1 ash, previously only generically inferred by heat effects on both the victims’ skeletons^13–16^ and the vitrified brain^17,64^, is now recorded at minimum temperature of > 550 °C by high reflectance values in polymodal charcoal datasets, whereas all other paleo-thermal data from the rest of the pyroclastic sequence indicate lower temperatures of diachronic processes related to the later burial of the town^34,36,37,65,66^.

Dilute PDCs are characterized by high air-entrainment coefficients^27,67^, therefore the recorded high temperature of S1 cannot be explained by a dilute current generated at vent and propagating as a surge for 7 km along the Vesuvius slope. Instead, we propose a new interpretation for the first Herculaneum event as an ash cloud surge detached from nearby high-concentration pyroclastic currents, as occurred at Unzen in 1991^7,68^ and Volcan de Fuego in 2018^11^. High-concentration pyroclastic currents, especially where valley confined, can maintain very high temperatures for kilometers from the vent as air entrainment is very limited (e.g.,^20–22^), whereas the overriding ash clouds may also maintain a similar high temperature for as long as they are supplied with mass and heat from below^23^. However, as soon as the ash cloud detaches from the basal high-concentration current and becomes an independent dilute surge, the temperature rapidly drops due to fast air entrainment and heat exchange promoted by the fine grain size of the pyroclasts.

**Figure 3:**
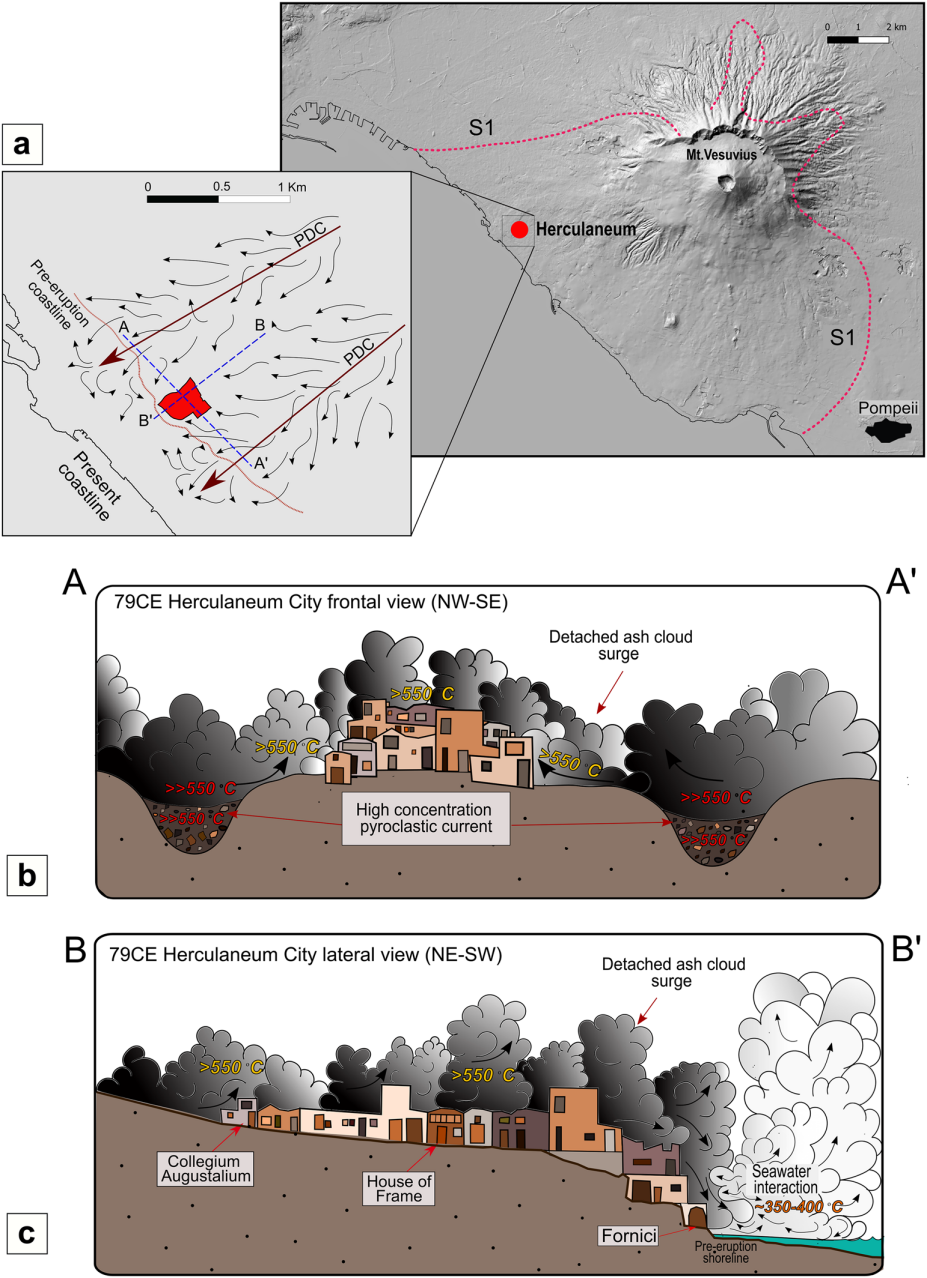
Scenario of the S1 ash cloud surge entrance in Herculaneum. (**a**) DTM (20 m) map of Vesuvius volcano (Qgis Software version 3.18 Zurich) with S1 areal distribution according to^30^ and location of Herculaneum. Inferred direction of the valley-confined pyroclastic flows (dark red straight arrows) and associated ash cloud surges (thin and curved black arrows) that reached Herculaneum and the pre-eruption shoreline (thin red line). (**b**) A–A′ transversal reconstruction (not to scale) of the ash cloud detachment from valley-confined high concentration PCs that acted as the high temperature (>> 550 °C) source of the ash cloud. (**c**) B–B′ longitudinal reconstruction (not to scale) of the S1 ash cloud engulfing the city at T > 550 °C and its interaction with seawater along the pre-eruptive shoreline, rapidly slowing the velocity and reducing the temperature (350–400 °C).

Herculaneum was built front facing the seashore on a relief some 10–15 m higher than sea level (Fig. 1), therefore on a topographic high likely sided north and south by valleys along which the denser parts of the pyroclastic currents would have been confined and presently buried underneath the modern town (Fig. 3a, c).

The most likely scenario for S1 at Herculaneum is, in our interpretation, that of an ash cloud decoupled^2^ from its parent high concentration valley confined pyroclastic current just in the proximity of the city, so that the ash cloud could form at high temperature (Fig. 3a–c). A similar scenario was reconstructed for the early phases of the Fogo A eruption (Azores)^69^.

Once crossed the city, 200 m downstream respect to the Decumanus Maximus, the S1 ash cloud surge jumped onto the beach and into the waterfront chambers (Fornici, Fig. 1), where it instantly killed the people who had taken refuge there^13^. The thermal effects detected on the victims’ bones found in the Fornici^13–16^, well match with the > 550 °C ash-cloud temperature measured upstream at the Collegium Augustalium and the Decumanus Maximus. The scattered preservation of bone collagen does not appear to be evidence of a low temperature of the ash cloud surge as claimed by some author^33^, but it seems to be related to the amount of heat transfer the victims' bodies and bones were exposed to during the short-lived ash cloud event. Actually, the greater or lesser extent of heat effects on the skeleton, or even on a single bone element, has been shown to be closely related to the lesser or greater crowding of victims inside the Fornici, and also amount of fleshy mass present in different anatomical districts, even at the level of a single bone^15,16^. However, the persistence of proteins such as collagen and other organic components of bones at Herculaneum is most likely independent by exposure to more or less intense heat but can be rather correlated to the burial environment in which the victims’ skeletons were embedded until their discovery after about 2000 years^70^. At Herculaneum, after sudden death and rapid thermally induced soft tissue vanishing, the skeletons were buried in an alkaline, anoxic soil permanently waterlogged^70^, environment able to inhibit chemical changes from microbial attack^71,72^ thus allowing long-term survival of organic matter in the bone.

The average lower temperature between 325 and 350 °C (Table 1) detected by charcoal at Fornici may be explained by the fast heat exchange due to the interaction of the ash cloud with both human bodies and the nearby seawater. Direct observations of pyroclastic currents entering the sea after travelling along the slopes of stratovolcanoes (e.g.,^73,74^) show that they rapidly inflate and cool due to entrainment of seawater. This effect has been documented at the nearby Villa dei Papiri^37^, and it is in agreement with well documented examples, including the 1902 ash surge that destroyed Saint Pierre, Martinique^4,5^ and the ash surges of Secche di Lazzaro, Stromboli^75^, where the combination of topographic effects and seawater engulfment at the coastal surge-water interaction, promoted the sudden surge expansion and even the backflow uphill of the cooled and dilute surge. A further cause of the lowering of the temperature of the deposit embedding the bodies of the victims in the Fornici could be the bodies themselves, as a direct source of a large amount of water vapor produced by the vaporization and rapid disappearance of soft body tissues induced by exposure of a huge number of victims to extreme heat^15,16^, environment presumably characterized by a higher temperature than that of the deposit itself.

We therefore interpret the first ash cloud surge S1 to have been very short-lived, reaching the coast and the Fornici still at > 500 °C, leaving almost no deposit but killing the people there^13,14^. The interaction of the ash cloud surge and seawater caused the surge inflation and the settling of cooled ash immediately after, which then embedded the skeletons of the people already killed instantly by the extreme heat (Fig. 3b A–A′ profile). This interpretation explains the apparent disagreement of recorded temperatures at the seashore and also the difference in thickness of S1, which is maximum 20 cm thick in town, whereas it reaches 50 cm along the pre-eruption coast (up to 150 cm in the Fornici according to^30^), where deposition was controlled by the slowed and cooled water-mixed ash cloud.

The results of this study bear unprecedented implications for the mitigation of volcanic risk at Vesuvius and possibly elsewhere. The red zone at Vesuvius, where full evacuation of ca 700.000 people is planned in case of a future eruption^76^ was designed based on the probability of PC invasion derived from the geological record^77^. While this is certainly the goal to be achieved, it remains uncertain whether the progression of the volcanic unrest will allow enough time to reach the expected full evacuation prior to eruption^78^. In addition, Plinian ignimbrites^76^ from directional and partial collapses of the eruptive column flowing confined along valleys and prone to ash cloud detachments, are more likely respect to axisymmetric caldera-forming ignimbrite^79^ from PCs covering all at once the entire red zone^77^. Given these premises, we suggest that the edifices within the red zone irrespective of the need to evacuate all people before the eruption, should be reinforced to be able to shelter people from the thermal impact of ash cloud surges in case full evacuation is not achieved on time. In facts, while zones exposed to high dynamic pressure of high concentration and high velocity PCs will inevitably see the collapse of edifices and structures with very little chances of survival, other zones may be impacted by short-lived detached ash clouds where potential for survival critically depends on the ability of shelters to prevent infiltration of the hot dusty gas. This could allow people who may not had the chance to evacuate earlier to survive and wait for rescue or be able to leave before other PCs may impact the area.

## Materials and methods

### Sample preparation procedure for reflectance analysis

Charcoal samples preparation and Reflectance analysis were performed at A.L.B.A. Laboratory at Roma Tre University, Rome. Samples were gently cleaned of ash particles and set in an epoxy resin and hardener mixture. After 48 h, they were polished with carborundum papers of different grit grade (250, 500, 1000) and subsequently polished with alumina powders of decreasing grain size (1, 0.3, 0.1 μm). Samples were analyzed under oil immersion with a Zeiss Axioskop 40 A pol microscope-photometer system (MPS system) equipped with a tungsten-halogen lamp (12 V, 100 W), an Epiplan-Neofluar 50 × /1.0 oil objective, using filtered 546 nm incident light.

Mono-crystalline prisms for the calibration of the reflection-photometer were used prior to performing reflectance measurements by means of the MPS 200 system of J&M to guarantee a calibration over a wide range of thermal maturity: spinel (Ro = 0.426), sapphire (Ro = 0.595), yttrium–aluminium-garnet (Ro = 0.905), and gadolinium-gallium-garnet (Ro = 1.726).

The instrument calibration was repeated after the measurement of each sample in order to maintain the maximum precision and the determination coefficient (R2), of the regression line (based on three standards) equal to or greater than 0.99998. For a good statistical representativity, a minimum of 50 reflectance measurements were performed on each charcoal sample, selecting only fragments whose surfaces were unaltered.

## Supplementary Information

Supplementary Information.

## Data Availability

The online version contains Supplementary Material available. In case of further information contact the corresponding author A.P.
