# Supplementary material for: A new hazard scenario at Vesuvius: deadly thermal impact of detached ash cloud surges in 79CE at Herculaneum
Source: Sci Rep. 2023 Apr 6;13:5622. doi: 10.1038/s41598-023-32623-3 (PMC10079856; doi:10.1038/s41598-023-32623-3)
Supplement: Supplementary file 1 — Supplementary Information. [file 41598_2023_32623_MOESM1_ESM.pdf]

Supplementary Materials

Manuscript: "A new hazard scenario at Vesuvius: deadly thermal impact of detached ash cloud surges in 79CE at Herculaneum"

Alessandra Pensa*[1,2], Guido Giordano[1], Sveva Corrado[1], Pier Paolo Petrone[3]

*Corresponding author. Email: alessandra.pensa@isprambiente.it

Supplementary Table1.1. Descriptive table of the 40 samples collected for this study at Herculaneum.

SITE		DEPOSIT	SAMPLE		IMAGE
1.Collegium Augustalium			Charred wood fragments from the bed frame	ERC-5A	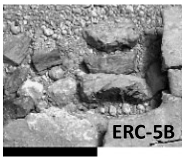
				ERC-5B	
				ERC-19A	
				ERC-19B	
			Charred wood fragments from support beam within the chamber	ERC-7A	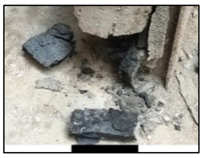 ERC-8A
				ERC-7B	
				ERC-8A	
				ERC-8B	
				ERC-16	
				ERC-17	
				ERC-18	
		Fine ash deposit encasing the skeleton	Charred wood fragments from the skeleton	ERC-6	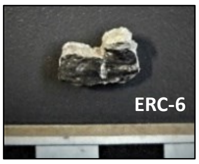
2.Decumanus Maximus		Massive pyroclastic deposit	Charred wood fragments from a door beam	ERC-54A	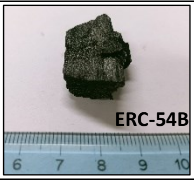
				ERC-54B	
		Fine ash deposit (from Surge S1)	Charred wood fragments sieved from the ash deposit	ERC-55	
3.Stores/ Houses	A	Massive pyroclastic deposit	Charred wood fragments	ERC-9A	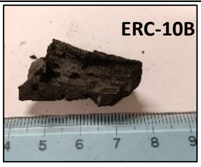
	B	Massive pyroclastic deposit	Charred wood fragments	ERC-10A	
				ERC-10B	
				ERC-12	
	C	Massive pyroclastic deposit	Charred wood fragments from the upper floor	ERC-13A	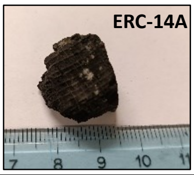
				ERC-13B	
				ERC-14A	
	D	Massive pyroclastic deposit	Charred wood fragments from the upper floor	ERC-15A	
				ERC-15B	
4.House of the Frame		Fine ash deposit	Charred seed	ERC-31A	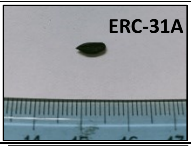
5.Fornici/ Pre-eruption beach		Fine ash deposit	Charred wood fragments from ash filling a victim's skull	ERC-20	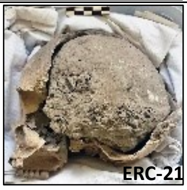
				ERC-21	
				ERC-22	
				ERC-23	
		Fine ash deposit	Charred wood fragment	ERC-21	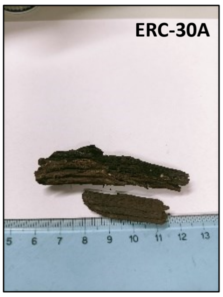
		Fine ash deposit	Charred wood fragment	ERC-22	
		Fine ash deposit	Charred wood fragment	ERC-23	
		Fine ash deposit	Charred wood fragment	ERC-29A	
		Fine ash deposit	Charred wood fragment	ERC-30A	
		Fine ash deposit	Charred wood fragment	ERC-32	
		Fine ash deposit	Charred cork	ERC-33	
		Fine ash deposit	Charred wood fragment	ERC-34	
		Fine ash deposit	Charred bamboo fragment	ERC-35	
		Fine ash deposit	Charred wood fragment	ERC-36	
		Fine ash deposit	Charred wood fragment from ribs	ERC-53	

Supplementary Table 1.1 In table are reported in detail all the samples main information: Location divided by sampling site, the deposit source, the samples name, description, and representative image.

Supplementary Text 1.2 Choice of the reference Charcoal Reflectance Analysis and pyrolysis curve for the Herculaneum samples.

Based on previous Charcoal Reflectance studies first undertaken during the 1970s and 80s ([1,2]), [3,4] were among firsts to investigate the use of reflectance analysis on wood charred by pyroclastic flows to assess their emplacement temperature.

The experiments carried out on pyrolysis of *Sequoia Sempervires* and *Gandoderma fungus* samples, burned in anoxic condition in a pre-set oven at different increasing temperatures and exposure time, demonstrated that both wood and fungus, despite belonging to different species, display increased reflectance degree ([3,4]). In details the exposure time versus reflectance percentage graphs highlighted that reflectance rises rapidly within the first hour for temperature below 450 °C and then become almost steady, while for higher temperature reflectance degree continues to increase with slower rate up to 24h.

Further experiments were carried out by [5–13] using different plants (*Pinus sylvestris, Quercus robur, Betula nana,Betula pendula, Picea mariana, Picea glauca, Betula papyrifera, Populus tremuloides, Rhizophora apiculate, Triticum aestivum, Pisum sativum and Helianthus annuus)* to assess temperature of recent and ancient wildfires, archaeological burned sites and wood charred by pyroclastic processes.

Despite the variation in taxa, the direct relationship between increasing temperature and reflectance degree remained comparable in all experiments. However, an aspect that must be considered to distinguish the experiments is the heating protocol adopted.

Pyrolysis tests present in literature, can be subdivided into two main heating procedures: 1. Low Heating Rate with long exposure time (>60min) at different temperatures and/or using progressive heating steps starting from ambient temperature to pre-selected final temperatures; 2. High Heating Rate that consists in placing the wood samples directly in a pre-heated oven for a short exposure time (60min) at pre-selected high temperature (800-1200°C). The application of different heating protocols revealed different responses in terms of mass loss depending on the taxonomic identity. In the experiments carried out by [6] wood fragments belonging to

gymnosperms (seed plants not flowering) as pine, sequoia and spruce trees and to angiosperms (flowering seed plants) as oaks, birch and mangrove trees displayed differences in mass loss during carbonification. With both protocols (LHR and HHR) and up to 300 °C, oak, and pine fragments (as representative samples of angiosperms and gymnosperms respectively) change slightly respect to their row wood status in terms of texture. Both display same dark colour and rapid mass loss (average 60%) but with oak fragment showing a greater mass loss compared to pine sample; contrary this ratio is reversed for temperatures above 350 °C. The difference in mass loss percentage also appears to be affected by the choice of heating procedure as with HHR the mass loss increases of 5-10% respect to LHR.

The difference between gymnosperms and angiosperms during carbonification processes reflects also in a discrepancy of reflectance signal at same temperature condition. As demonstrated by [6] at equal carbonification temperature angiosperms (oak wood) display higher reflectance percentage than gymnosperms (pine wood). Difference between conifers and broad-leaved trees reflectance data was also highlighted by [10] in the temperature assessment of the 1.8 ka Taupo ignimbrite, New Zealand and by [9] with pine tree and mangrove tree pyrolysis experiments.

Supplementary Figure 1.3. Pyrolysis curves present in literature

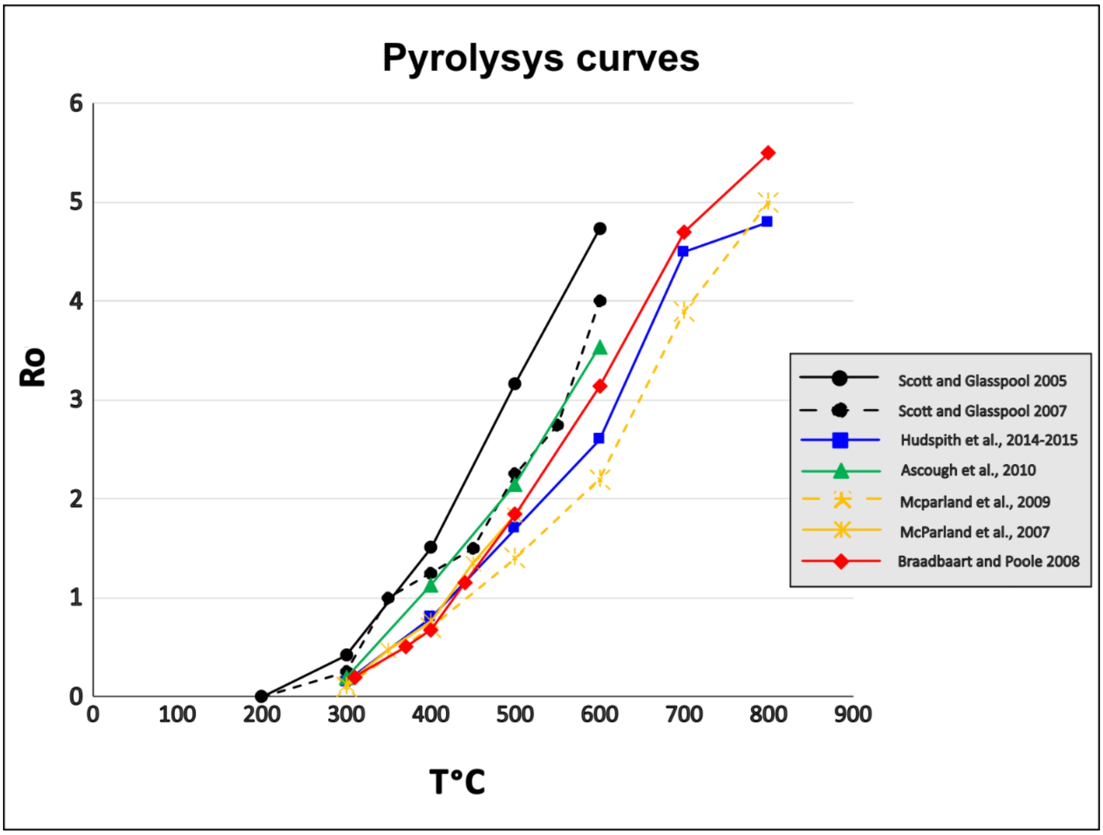

Supplementary Figure 1.3 Conversion of reflectance measurements (Ro) into temperatures

Representation of seven pyrolysis curves present in literature of [3] (solid black line with circles), [4] (dashed black line with circles), [11,12] (solid blue line with squares); [9] (green solid line with triangles), [7] (dashed yellow line with stars), [8] (solid yellow line with stars) and [6] (solid red line with diamonds). Curves were redraw based on published data.

Supplementary Table 1.4 Pyrolysis experiments adopting different charring procedures and wood type

	WOOD TYPE	FURNACE T CONDITION	TIME	T RANGE	NOTE
**SCOTT & GLASSPOOL 2005-2007**	Sequoia<br>Fungus	preheated	168h	200-900°C	
**MCPARLAND ET AL 2007**	Ferns<br>Sequoia	preheated	1h	200-1000°C	
**BRAADBAART & POOLE 2008**	Pine<br>Birch<br>Oak	HHR: preheated<br><br>LHR: From ambient T	1h<br><br>2h	300-1200°C	
**MCPARLAND ET AL 2009**	Oak	preheated	5h	300-800°C	
**ASCOUGH ET AL 2010**	Pine<br>Mangrove	From ambient T	1h	300-600°C	
**HUDSPITH ET AL 2010**	Sequoia ([3,4])	preheated	168h ([3,4])	200-900°C ([3,4])	
**HUDSPITH ET AL 2014-2015**	Birch<br>Aspen<br>Spruce	preheated	1h	300-800°C	Pyrolysis curve combined

Supplementary Table 1.5. reports the principal pyrolysis experiments conducted in the last 20 years using different wood types, heating procedures, heat exposure times and maximum pre-set temperature. Here are reported the principal

pyrolysis experiments conducted in the last 20 years using different wood type, heating procedures, heat exposure time and maximum pre-set temperature. As reported in [14] the comparison of these pyrolysis curves highlights the same proportional relationship between rising temperature and increasing Reflectance degree but with differences in slope for temperature > 300 °C. It follows that the choice of the curve to use for the conversion of the reflectance degree into temperature is extremely important as, above Ro=1, the same reflectance value can be converted in a wide range of temperature depending on the curve selected.

Supplementary Fig 1.5 Flow diagram describing the four filters applied to select the most appropriate pyrolysis curve

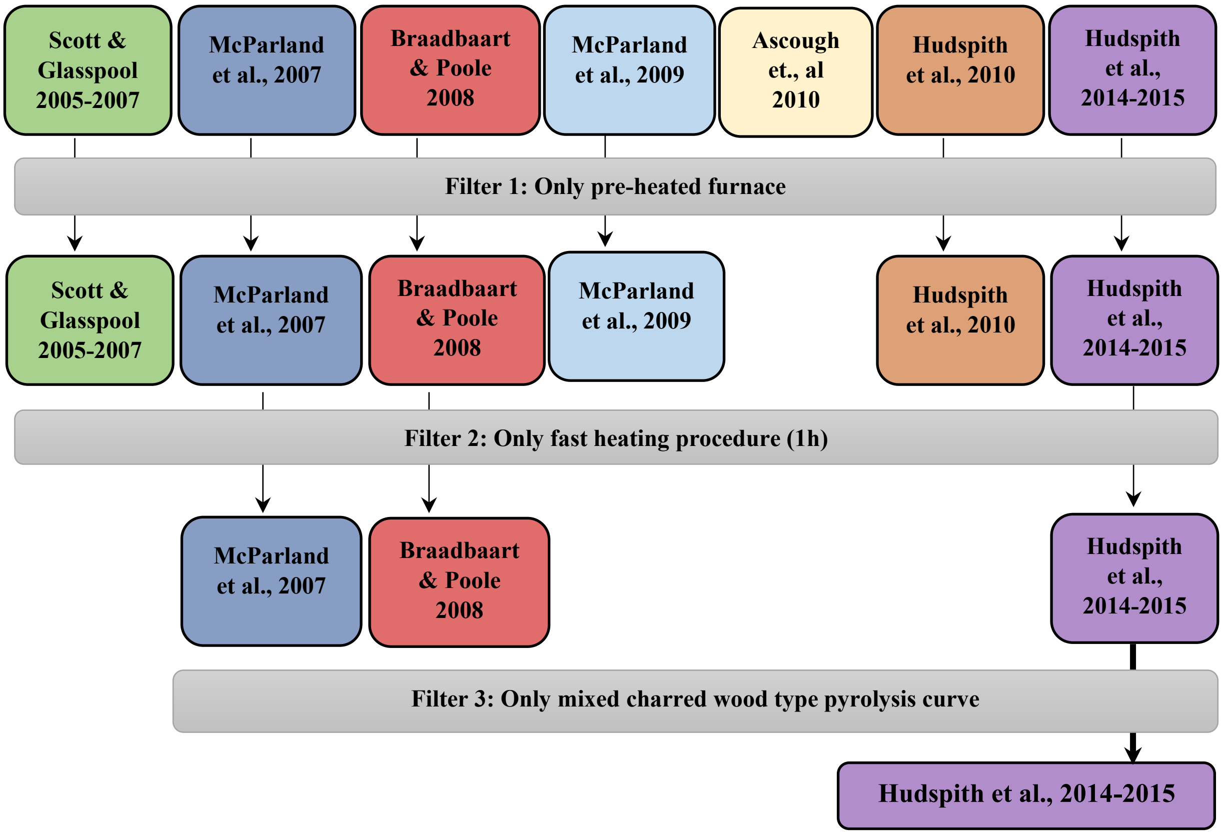

Supplementary Figure 1.5 To better choose the most appropriate curve for the forty charred wood samples collected in Herculaneum we applied three filters according to the nature and dynamics of the volcanic process and according to the wood type. As displayed in the flow diagram, the first filter consisted in excluding experiments not conducted in pre-heated furnace but starting from ambient temperature. This is because the thermal impact of a pyroclastic current is instantaneous at high temperature and does not increase with time. For the same reasons, we also preferred high heating rates (Supplementary Table 1.4). The third filter applied is related to the pureness of the pyrolysis curve. As our samples reflect a range of wood types, we selected the pyrolysis curves representative of multiple charring experiments mixing conifer and broadleaves ([11,12]). The result of this filtering process elects the [11] curve as the most appropriate pyrolysis curve to use for the conversion of Reflectance degree into temperature values for the samples collected at Herculaneum.

Supplementary material Reference

1. Correia & Maury. Mesure, par leur pouvoir réflecteur, des températures de carbonisation des bois fossilisés dans les formations volcaniques. *Bull. Cent. Rech. Pau* **8**, 527–536 (1974).

2. Bustin, R. M. & Guo, Y. Abrupt changes (jumps) in reflectance values and chemical compositions of artificial charcoals and inertinite in coals. *Int. J. Coal Geol.* **38**, 237–260 (1999).

3. Scott, A. C. & Glasspool, I. J. Charcoal reflectance as a proxy for the emplacement temperature of pyroclastic flow deposits. *Geology* **33**, 589–592 (2005).

4. Scott, A. C. & Glasspool, I. J. Observations and experiments on the origin and formation of inertinite group macerals. *Int. J. Coal Geol.* (2007) doi:10.1016/j.coal.2006.02.009.

5. Braadbaart, F., Boon, J. J., Veld, H., David, P. & van Bergen, P. F. Laboratory simulations of the transformation of peas as a result of heat treatment: changes of the physical and chemical properties. *J. Archaeol. Sci.* **31**, 821–833 (2004).

6. Braadbaart, F. & Poole, I. Morphological, chemical and physical changes during charcoalification of wood and its relevance to archaeological contexts. *J. Archaeol. Sci.* **35**, 2434–2445 (2008).

7. McParland, L. C., Collinson, M. E., Scott, A. C. & Campbell, G. The use of reflectance values for the interpretation of natural and anthropogenic charcoal assemblages. *Archaeol. Anthropol. Sci.* (2009) doi:10.1007/s12520-009-0018-z.

8. McParland, L. C. *et al.* Ferns and fires: Experimental charring of ferns compared to wood and implications for paleobiology, paleoecology, coal petrology, and isotope geochemistry. *Palaios* **22**, 528–538 (2007).

9. Ascough, P. L. *et al.* Charcoal reflectance measurements: Implications for structural characterization and assessment of diagenetic alteration. *J. Archaeol. Sci.* **37**, 1590–1599 (2010).

10. Hudspith, V. A., Scott, A. C., Wilson, C. J. N. & Collinson, M. E. Charring of woods by volcanic processes: An example from the Taupo ignimbrite, New Zealand. *Palaeogeogr. Palaeoclimatol. Palaeoecol.* (2010) doi:10.1016/j.palaeo.2009.06.036.

11. Hudspith, V. A., Belcher, C. M. & Yearsley, J. M. Charring temperatures are driven by the fuel types burned in a peatland wildfire. *Front. Plant Sci.* **5**, 1–12 (2014).

12. Hudspith, V. A., Belcher, C. M., Kelly, R. & Hu, F. S. Charcoal reflectance reveals early Holocene boreal deciduous forests burned at high intensities. *PLoS One* **10**, (2015).

13. Hudspith, V. A. & Belcher, C. M. Observations of the structural changes that occur during charcoalification: implications for identifying charcoal in the fossil record. *Palaeontology* **60**, 503–510 (2017).

14. Pensa, A., Capra, L., Giordano, G. & Corrado, S. Emplacement temperature estimation of the 2015 dome collapse of Volcán de Colima as key proxy for flow dynamics of confined and unconfined pyroclastic density currents. *J. Volcanol. Geotherm. Res.* **357**, 321–338 (2018).
